# Determination of endogenous sphingolipid content in stroke rats and HT22 cells subjected to oxygen-glucose deprivation by LC‒MS/MS

**DOI:** 10.1186/s12944-022-01762-3

**Published:** 2023-01-25

**Authors:** Keqi Zeng, Xin Zhou, Wanyi Liu, Cong Nie, Yingfeng Zhang

**Affiliations:** Department of Pharmaceutics, College of Chinese Materia Medica, Guangzhou University of Chinese Medicine, Guangdong 51006 Guangzhou, China

**Keywords:** Ischemic stroke, Content determination, Sphingolipids, Endogenous, OGD/R-Induced, Liquid chromatography, Mass spectrometry

## Abstract

**Background:**

Stroke is the leading cause of death in humans worldwide, and its incidence increases every year. It is well documented that lipids are closely related to stroke. Analyzing the changes in lipid content in the stroke model after absolute quantification and investigating whether changes in lipid content can predict stroke severity provides a basis for the combination of clinical stroke and quantitative lipid indicators.

**Methods:**

This paper establishes a rapid, sensitive, and reliable LC‒MS/MS analytical method for the detection of endogenous sphingolipids in rat serum and brain tissue and HT22 cells and quantifies the changes in sphingolipid content in the serum and brain tissue of rats from the normal and pMCAO groups and in cells from the normal and OGD/R groups. Using sphingosine (d17:1) as the internal standard, a chloroform: methanol (9:1) mixed system was used for protein precipitation and lipid extraction, followed by analysis by reversed-phase liquid chromatography coupled to triple quadrupole mass spectrometry.

**Results:**

Based on absolute quantitative analysis of lipids in multiple biological samples, our results show that compared with those in the normal group, the contents of sphinganine (d16:0), sphinganine (d18:0), and phytosphingosine were significantly increased in the model group, except sphingosine-1-phosphate, which was decreased in various biological samples. The levels of each sphingolipid component in serum fluctuate with time.

**Conclusion:**

This isotope-free and derivatization-free LC‒MS/MS method can achieve absolute quantification of sphingolipids in biological samples, which may also help identify lipid biomarkers of cerebral ischemia.

**Graphical Abstract:**

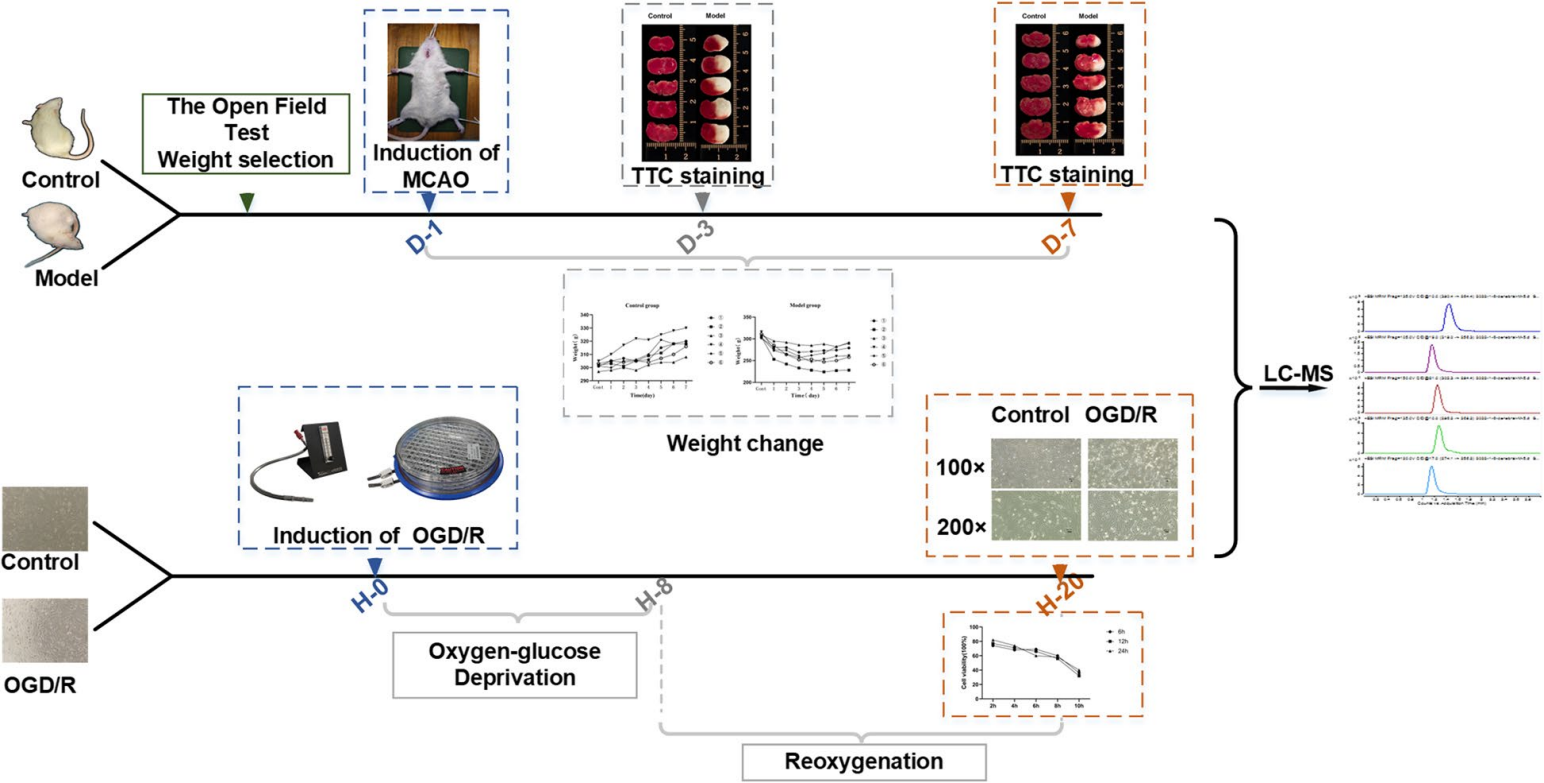

**Supplementary Information:**

The online version contains supplementary material available at 10.1186/s12944-022-01762-3.

## Introduction

Stroke is a destructive cerebrovascular disease that reduces or even interrupts cerebral blood flow due to occlusion or rupture of cerebral blood vessels, which can cause physical disability and multiple functional impairments, of which more than 80% are ischemic strokes [1], characterized by high morbidity, high mortality, high disability, and a high recurrence rate [2]. Stroke is one of the major causes of human disability and death and poses a serious public health threat [3]. Currently, there are limited methods for the treatment of ischemic stroke. Mechanical thrombectomy or intravenous thrombolysis is often used alone or in combination, and the combination of thrombectomy and bridging therapy has been reported to restore normal cerebral blood flow [4, 5]. Reducing cell death by repairing or establishing alternative neural pathways and synapses to repair nerves or intervene in the ischemic cascade is also meaningful to explore [6]. The development of neuroprotective drugs for stroke is a slowly evolving process [7], and it is necessary to explore new targets for stroke treatment.

The brain is highly enriched in lipids, which are significant parts of cell membranes, play an important role in intracellular and intercellular signaling pathways in brain cells, and maintain the homeostasis of the intracellular environment. Lipid rafts are an important component of cell membranes, with sphingolipids and cholesterol being the main components of lipid rafts. The maintenance of nervous system function depends partly on changes in lipid raft content [8]. Studies have shown that disorders of lipid metabolism and changes in lipid rafts are closely related to neurodegenerative diseases such as Alzheimer's disease (AD) [9] and stroke [10]. Strengthening lipid research can help unlock the potential of lipids as disease biomarkers.

Sphingolipids are the main components of cellular myelin. Sphingolipids such as ceramide, sphingosine, and sphingosine-1-phosphate play essential roles in signal transduction, cell proliferation, differentiation, and apoptosis [11]. Ceramidase hydrolyses ceramides to sphingosine. Sphingosine is an intracellular signaling lipid that is related to the release of neurotransmitters, participates in changes in cell growth and acts as a bioactive lipid against oxidative stress damage [12]. Sphingosine-1-phosphate (S1P), known as “bioactive sphingolipid”, is related to enhancing cell proliferation and differentiation and regulating cellular physiological and pathological processes [13]. The brain is the organ with the highest concentration of S1P [14]. Under pathological conditions such as stroke and inflammation, the concentration of local S1P and corresponding sphingolipids changes significantly [15, 16]. It is speculated that changes in the content of sphingosine components such as S1P can indicate the occurrence of stroke and other diseases.

Quantitative detection of endogenous components such as sphingolipids is a major challenge due to the presence of a large number of isomers, difficulty in obtaining a blank matrix, low natural abundance, and poor ionization efficiency. The determination of endogenous components mostly adopts the matrix substitution method, substitution analyte method, background subtraction method, standard addition method, and so on [17–19]. However, for biological samples with complex components, the greatest challenge is the difficulty of finding a widely recognized and suitable blank matrix (without the analyte) or an ideal substitute for the matrix [20]. The method of obtaining tissue substitutes using stable isotopes or structural derivatization makes them highly similar to real biological matrices, but the operation is cumbersome and costly, so clinical applications are limited. The background subtraction method detects the real analyte based on the real matrix, and the results are more accurate and intuitive. The natural abundance of sphingolipids is very low. To date, the ideal relative quantitative detection methods are UHPLC‒MS/MS [21], UFLC‒MS/MS [22], etc. However, most of them require derivatization treatment, which is cumbersome, time-consuming, and has low recovery. Sphingolipid absolute quantification is of great significance for the diagnosis and prognosis evaluation of common clinical diseases such as stroke by combining appropriate matrix and analytical methods.

Based on the possible important roles of sphingolipids in the pathogenesis, clinical diagnosis, and prognosis evaluation of ischemic stroke, this paper proposes to establish a rapid, sensitive, and reliable LC‒MS/MS analysis method combined with the matrix substitution method and background subtraction method to detect changes in the content of sphingolipids such as S1P in serum and brain tissue of the normal group and permanent middle cerebral artery occlusion (pMCAO) model rats and in HT22 cells of the normal group and oxygen–glucose deprivation (OGD/R) group. It also revealed the role of endogenous sphingolipids in stroke injury and their potential as possible diagnostic markers.

## Materials and methods

### Chemicals and reagents

D-erythro-1-sphingosine phosphate (sphingosine-1-phosphate, S1P) was purchased from GlpBio (Shanghai, China). D-Sphingosine (sphingosine (d17:1)) was acquired from Avanti Polar Lipids (Alabaster, AL). DL-erythro/threo sphinganine (d16:0), sphinganine (d18:0), and phytosphingosine were obtained from Cayman (MI, USA). Tetrazolium Red was purchased from Macklin Inc. (Shanghai, China). Cell-Counting Kit-8 was obtained from Biosharp (Guangzhou, China), and isoflurane was purchased from MCE (Shanghai, China). Deionized water was prepared from a Millipore Milli-Q purification system (Bedford, MA, USA). Bovine serum albumin (BSA) was obtained from Sigma (USA). All chemicals used were of pure analytical grade.

### Laboratory animals and animal modeling

Male SD rats weighing 300 to 350 g were obtained from the Laboratory Animal Center of Guangzhou University of Chinese Medicine (approval number: SCXK (YUE) 2019–0047). Animals were raised in the Laboratory Animal Center of Guangzhou University of Chinese Medicine (approval number: SYXK (YUE) 2018–0085). This animal experiment was approved by the Laboratory Animal Ethics Committee of Guangzhou University of Chinese Medicine. The experimental protocol was in accordance with the requirements of the use of Laboratory Animals and Institutional Animal Care and Use Committee of Guangzhou University of Chinese Medicine (Record number: 20210323002).

Animal experiments were carried out on rats with no difference in behavior parameters in the open field test. Animals were randomly divided into two groups. Six rats from each group were taken for sample collection on the third and seventh days, and two rats from each group were randomly selected for model validation. Rats were group-housed in temperature-controlled rooms on a 12-h light–dark cycle and fasted for 12 h except for water before the experiment. The rat pMCAO model was prepared according to the Zea Longa suture-occluded method [23] and scored according to the Longa scoring method. Rats with scores of 0 and 4 were excluded when selecting the model rats. The two groups of animals were anesthetized with 2% isoflurane inhalation on the third day of modeling, and blood was taken from the inner canthus venous plexus. Then, serum was separated by centrifugation. On the seventh day, the rats were anesthetized using isoflurane and blood was collected from the abdominal aorta. After sampling, painlessly euthanized rats were euthanized by inhalation of overdosed isoflurane in accordance with animal ethics. The brain tissues were harvested on ice. Biological samples were immediately frozen in liquid nitrogen for 30 min and then stored at -80 °C.

### Cell culture and cell modeling

The HT22 mouse hippocampal neuron cell line was purchased from Shanghai Kanglang Biotechnology Co., Ltd. The cells were cultured at 37 °C in a humidified 5% CO_2_ incubator in DMEM (Gibco) containing 10% (*v/v*) fetal bovine serum and 1% penicillin/streptomycin. The culture medium was changed every 24 h, and the cells were digested with 0.25% trypsin for passaging when they reached 80% confluence. In all experiments, cells were seeded in 96-well plates/60 mm dishes and cultured for 24 h (1 × 10^4^ cells/well, *n* = 6; 2 × 10^6^ cells/dish, *n* = 6) before experimental treatment. The OGD/R model was established in HT22 cells: the culture medium was discarded and the HT22 cells were washed with PBS followed by the addition of glucose-free DMEM. Then, the medium was placed in a hypoxia chamber (95% N_2_,4% CO_2_,1% O_2_) (Stem Cell Technology, Vancouver, Canada) for 8 h. During the replication and reoxygenation process, the glucose-free DMEM was replaced with high glucose DMEM, and HT22 cells were then cultured in an incubator under normoxic conditions at 37 °C and 5% CO_2_ for an additional 12 h. After modeling, the cells in the culture dish were counted and stored at -80 °C, and the HT22 cells grown in 96-well plates were used for the cell viability test.

### Analytical procedure

#### Sample preparation

Six random SD rat brain tissues were accurately weighed and added to 4 °C normal saline (1 mL/g) in proportion, which was ground in a homogenizer at a frequency of 60 *Hz* for 3 min at 4 °C. Brain homogenate and serum(100 μL) and 1 × 10^6^ cells of the normal group and model group (*n* = 6), were combined with 300 μL of precooled extraction solution (chloroform: methanol = 9:1,*v/v*) (containing 0.01% BHT and 100 ng/mL internal standard), vortexed for 1 min, and left to stand at -20 °C for 1 h to further precipitate the protein. After centrifugation at 4 °C and 5000 × g for 20 min, all the supernatant was removed and dried under nitrogen flow at 40 °C, reconstituted with 100 μL precooled methanol, vortexed, and transferred to -20 °C for cryopreservation. Before injection, the sample was centrifuged for 10 min (10,000 ×g) at 4 ℃, which was repeated intermittently twice. The prepared supernatant was placed in a sample bottle containing an internal cannula for LC‒MS/MS analysis.

#### Stock and working solutions

The storage solution of S1P was configured by (DMSO/concentrated hydrochloric acid (100:2), *v/v*) at a concentration of 500 μg/mL. All the reference stock solutions except for S1P were prepared with chromatographic methanol at a concentration of 500 μg/mL and stored at -20 °C. During the experiment, the stock solution was further diluted with chromatographic methanol.

#### QC samples

A blank serum simulant was prepared by mixing BSA with normal saline at 4 ℃, resulting in a 4% BSA solution. QC samples of serum were prepared by adding the blank serum simulant to the diluted standard mixture (including internal standard) and then synchronized with the sample according to the “Sample Preparation” step. The normal group of brain tissue mixed homogenate 6 copies, each 100 μL, were added to a certain concentration of mixed reference (including internal standard), and cell samples of 1 × 10^6^ normal group (*n* = 6) were added with a certain concentration of mixed reference (including internal standard), and the samples were treated according to the “Sample Preparation” step. Finally, these samples were redissolved in methanol to obtain the standard substance of a range of concentrations. Quality control samples (QCs) were prepared at three concentration levels. All prepared solutions were stored in batches at -80 °C for use at different time points.

### Analysis by HPLC/MS

Chromatographic separation was achieved utilizing an Agilent ZORBAX SB-Aq column (2.1 × 100 mm, 3.5 μm, Agilent Technologies, Santa Clara, CA, USA) and an Agilent 1260 HPLC system (Agilent Technologies) with reversed-phase chromatography. The mobile phase consisted of methanol (A) and water (B) (85:15, *v/v*), both containing 0.1% formic acid and 5 mmol/L ammonium formate, where the flow rate was set at 0.3 mL/min with isocratic elution (3 min: 85% A). All samples were kept at 4 ℃ in sample trays before the analysis. A 5 µL aliquot of each sample was injected into the column, and the column temperature was maintained at 30 ℃. MS was performed using an Agilent 6460 triple quadrupole mass spectrometer (Agilent Technologies). Ions were detected using multiple-reaction monitoring mode (MRM). The electrospray ionization (ESI) source was operated at a 300 °C vaporizer temperature and a 350 °C capillary temperature in positive ionization mode with a spray voltage of 4000 V. The sheath gas and auxiliary gas were 45 and 15 psi, respectively. Nitrogen was used as the collision gas. Collision-induced dissociation (CID) was used with energies ramped from 10 to 40 eV, and the scan time was 0.1 s.

### Analytical validation

#### Specificity

Pure methanol, blank serum simulant, standard substance, serum and brain tissue samples obtained from animal experiments, and cell samples obtained from cell experiments were used for sample pretreatment according to the “Sample Preparation” step, and the samples were injected for determination to examine the method specificity.

#### Linearity and sensitivity

Quantitation of control samples and authentic samples was performed using an internal standard method. Calibration curves were established by employing a linear regression. The ratio of the peak area of the blank serum simulant solution plus the standard substance as the sample to the peak area of the internal standard was the Y-axis, and the concentration of the standard sample was the X-axis, which was expressed in the form of Y = aX + b, where a and b are constants.

The establishment of a quantitative calibration curve for brain tissue and cell samples: The known concentration was taken as the X-axis, and the ratio of the peak area of the quality control samples with different concentrations to the peak area of the internal standard was taken as the Y-axis to obtain the standard curve (Eq. 1). The b value (representing the endogenous lipid concentration contained in the matrix itself) was calculated from the intercept of this equation. Y1 (representing the peak area corresponding to the concentration of standards)was obtained by calculation, and the background deduction calibration curve (Eq. 3) was obtained by fitting Y1 and x. According to (Eq. 4), the absolute content of the components contained in each sample can be obtained.

1 $$Y=ax+b$$

2 $${Y}_{1}=Y-b$$

3 $${Y}_{1}={a}_{1}x+{b}_{1}$$

4 $$C=\left({Y}_{1}-{b}_{1}\right)/{a}_{1}$$

The linear range of the six concentration points of the standard curve of each component is shown in Table S-1. During the method validation, at least three qualified standard curves should be provided for inclusion in the statistics and cover the expected concentration range of the real samples. The LOD is defined as the lowest concentration (S/N ≥ 3) at which the detection can reliably distinguish the analyte peak signal from the background noise. The LOQ was defined as the lowest limit of detection of quantification (S/N > 10).

#### Precision and accuracy

According to the US Food and Drug Administration (FDA) guidelines, intra-assay and interassay precision and accuracy were assessed by analyzing QC standards at multiple different levels for three consecutive days. The concentrations of each component were as follows: sphinganine (d16:0) (1 ng, 3 ng, 1000 ng, 3000 ng), sphinganine (d18:0) (1 ng, 3 ng, 5000 ng, 15,000 ng), phytosphingosine (1 ng, 3 ng, 5000 ng, 15,000 ng) and S1P (4 ng, 10 ng, 125 ng, 375 ng) (*n* = 6).

#### Stability

These biological samples were pretreated according to the “Sample Preparation” step to prepare LQC (Low QC) and HQC (High QC) samples (*n* = 6) and investigated for stability together with the reference substance stock solution (0.5 mg/mL for each reference substance). (1) Short-term stability was determined as follows: QC samples and the reference substance stock solution were placed at room temperature for 48 h and -4 °C for 24 h; (2) Long-term stability was determined as follows: QC samples were kept frozen at -80 °C for 100 days, and the reference substance stock solution was placed at -20 °C for 120 days; (3) Freeze‒thaw stability was determined as follows: Both the QC sample and the reference substance stock solution underwent 5 freeze‒thaw cycles.

#### Matrix effects and sample recovery

The possible matrix effects and recoveries of the four components in serum, brain tissue, and cell samples were investigated. LQC, MQC (medium QC), and HQC samples were added to the mixed homogenate of brain tissue/mixed cell samples/blank serum simulant according to the “Sample Preparation” step (S1, *n* = 6). Six mixed homogenates of brain tissue/cell samples were used to prepare blank samples (S2, *n* = 6). To prepare six batches of QC samples of brain tissue and cells, a mixed reference solution equivalent to 80% (low), 100% (medium) and 120% (high) of the sample content was added before sample processing (S3, *n* = 6). LQC, MQC, and HQC were added before blank serum simulant treatment (S4, *n* = 6). Standards were obtained by dissolving LQC, MQC and HQC in methanol solution (S5, *n* = 6). Methanol diluted the standard solutions equivalent to 80% (low), 100% (medium), and 120% (high) of each component in brain tissue and cell samples (S6, *n* = 6) (all the above samples were added with 100 ng/mL Sphingosine (d17:1) (IS) with the time of QC except for sample 2. Sample 2 was spiked with sphingosine (d17:1) (IS) before sample processing). The samples were pretreated according to the “Sample Preparation” step and then subjected to LC‒MS measurement to obtain the ratio of the peak area of each component to the internal standard peak.

5 $$\textit{Ma}\text{trix effect}(\%)=\frac{\textit{S}\text{1 peak area/the peak area of the IS-S2 peak area/the peak area of the IS}}{\textit{S}\text{5 peak area/the peak area of }\text{the IS}}\times100$$

6 $$\text{Recovery }\textit{rate}\left(\textit{serum},\%\right)=\frac{\text{S4 peak area/the peak area of the IS}}{\text{S5 peak area/the peak area of the IS}}\times100$$

7 $$\text{Recovery }\textit{rate}\text{(}\textit{brain}\text{ tissue/}\textit{cell},\%)=\frac{\text{S3 peak are/the peak are of the IS-S2 peak area}}{\text{S6 peak area/the peak area of the IS}}\times100$$

### Statistical analyses

Agilent Mass Hunter Workstation Software was used to analyze all the chemicals (version B.05.00; Agilent Technologies, Inc.). Images were integrated after smoothing using a Gaussian function. The structures of the chemicals were created using ChemDraw 18.0 (PerkinElmer, Waltham, MS, USA). SPSS 20.0 software (SPSS, Chicago, IL, USA) was used for statistical processing, and all data are expressed as the mean ± standard deviation (SD). The independent-samples t test was used, and *P* < 0.05 indicated statistical significance (***P < 0.001, **P < 0.01, and *P < 0.05).

## Results

### Verification of the pMCAO rat model and OGD/R HT22 model

**Fig. 1:**
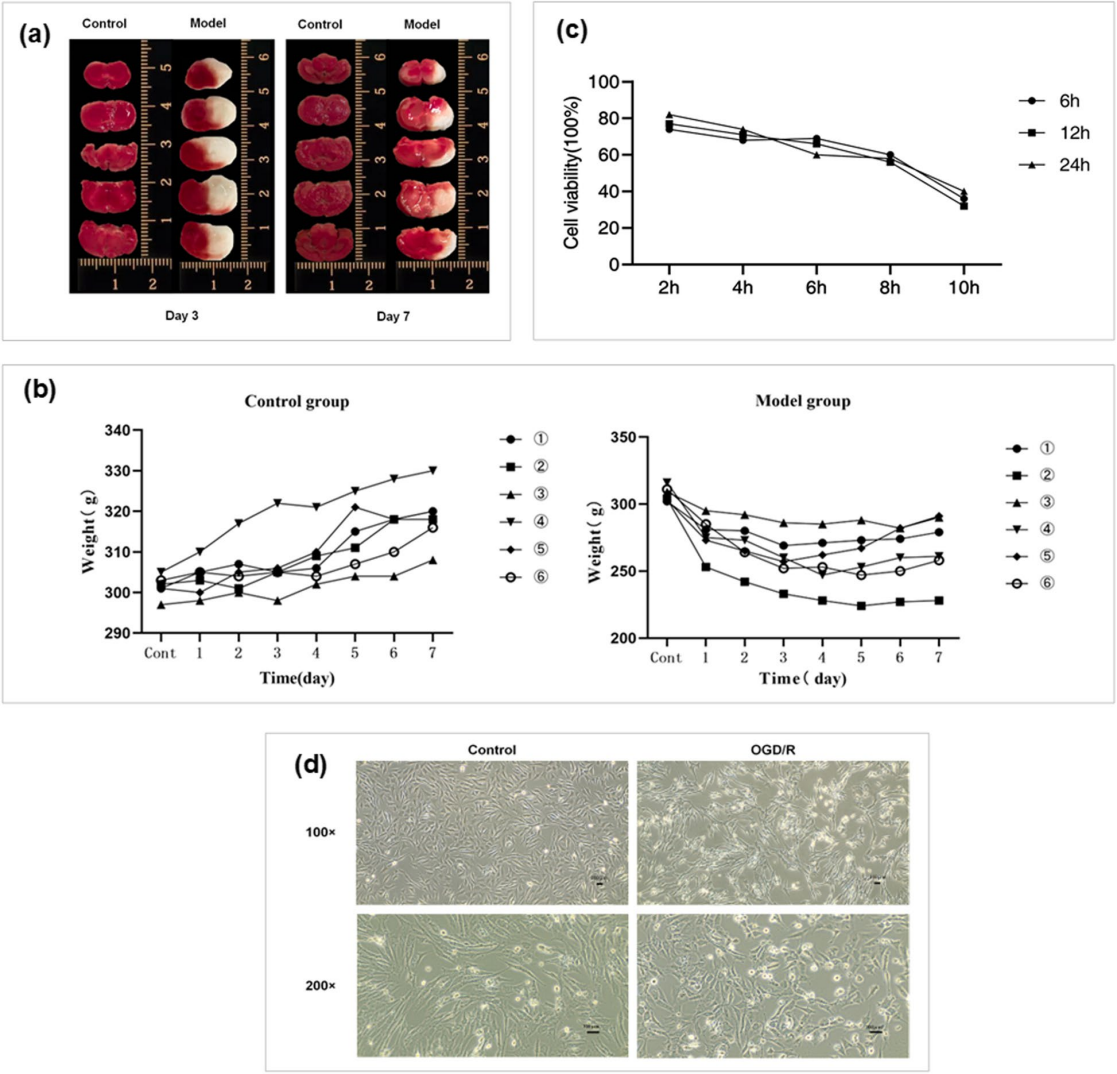
Verification of the pMCAO rat model and OGD/R HT22 model. **a** Determination of brain injury by TTC staining, **b** changes in weights of rats in normal and model groups, **c** cell viability detected by CCK-8, **d** inverted microscope to observe the morphological changes of HT22 cells after OGD/R injury

Rats were selected randomly from both the model and normal groups for TTC staining to verify the pMCAO model. Red areas represent noninfarcted tissue regions and white areas represent infarcted tissue regions (Fig. 1a). The infarct area was obvious, and the modeling was successful. Before the experiment, rats with no significant difference in the open field test and body weight were selected for modeling. The day of modeling was recorded as Day 0. The weights of the rats in each group following modeling were recorded. As shown in Fig. 1b, the weights of the rats in the normal group gradually increased over time, while the weights of the rats in the model group decreased significantly two days after modeling, then plateaued over time and recovered slightly on the seventh day.

To determine the optimal injury conditions for OGD/R injury, reoxygenation was performed for 6, 12, and 24 h following 2, 4, 6, 8, and 10 h of OGD. After modeling, the 96-well plates of each group were added to CCK-8 (10 μL/well). The optical density (OD) values were measured at 450 nm after incubation with CCK-8 reagent for 2 h at 37 °C. The blank group contained no cells but contained medium and CCK-8. Calculation: Cell viability (%) = (test OD value-blank OD value)/(control OD value-blank OD value) × 100%. The results (Fig. 1c) showed that the cell viability was 56% after hypoxia for 8 h and reoxygenation for 12 h, which was the best modeling condition for the OGD/R model of HT22 cells. At the same time, OGD/R induced morphological changes in HT22 cells. The cells in the normal group exhibited an elongated spindle-shaped appearance with clear outlines, and the protrusions were interwoven into a mesh and adhered. The cells of the OGD/R model group became round, the protrusions appeared shrunken, cytoplasmic condensation, and intercellular connections were greatly reduced, the cells were not tightly adherent, and part of the cell mass was sparsely shed (listed in Fig. 1d).

### Optimization of experimental conditions

The conditions optimization was mainly carried out from the choice of the column, the setting of different ratios of the organic phase and aqueous phase, liquid chromatography elution mode (isocratic elution and gradient elution), and pretreatment methods.

**Fig. 2:**
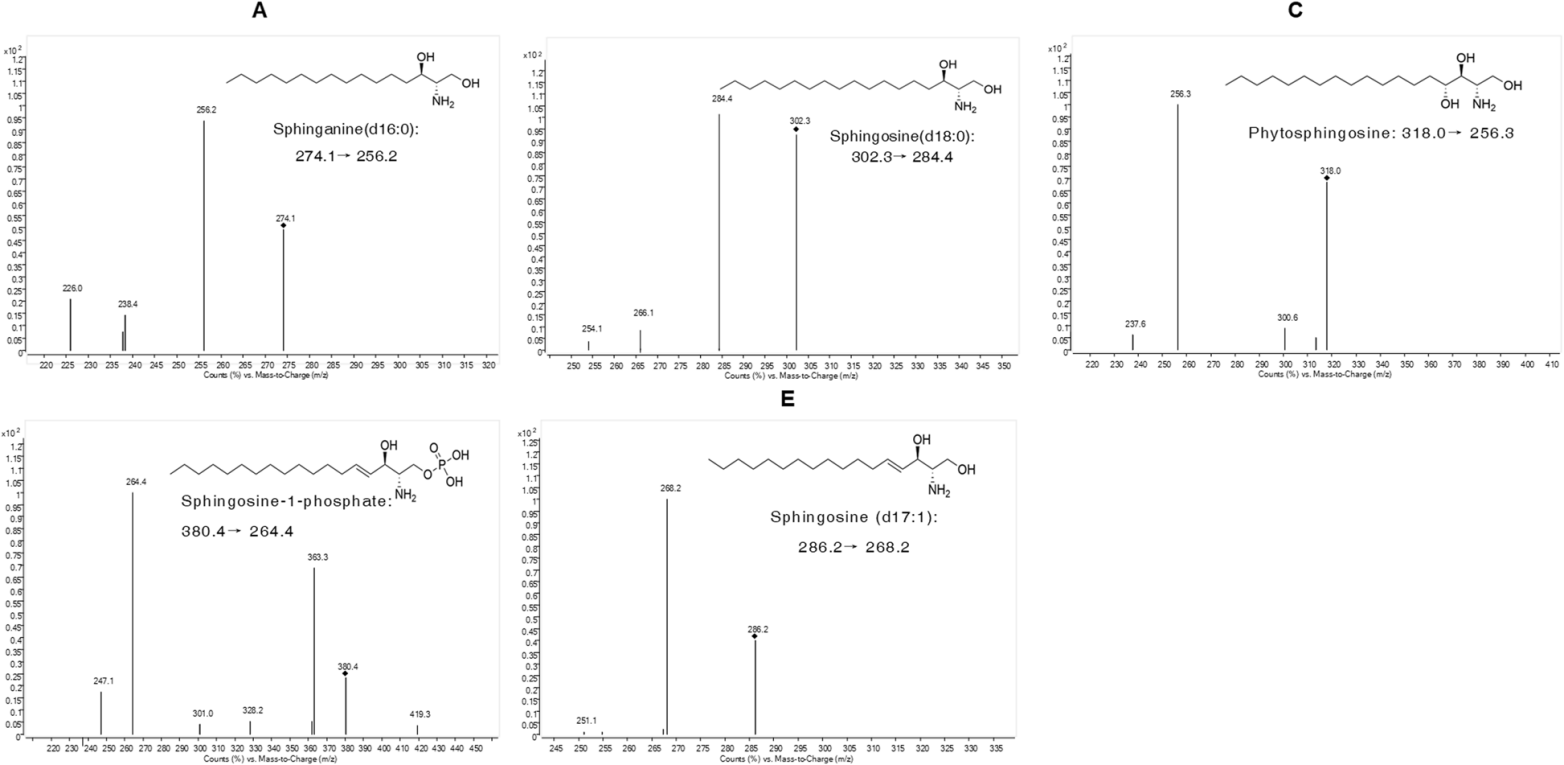
Product ion mass spectra of sphingosine (d16:0) (**A**), sphinganine (d18:0) (**B**), phytosphingosine (**C**), sphingosine-1-phosphate (**D**) and sphingosine (d17:1) (IS, **E**)

The choice of the column. In this study, according to the polarity and hydrophilicity of sphingosine, Thermo BDH HYPERSIL C18 (2.1 × 100 mm, 2.4 μm), Agilent ZORBAX SB-Aq C18 (2.1 × 100 mm, 5 μm) and Thermo ODS HYPERSIL C18 (4.6 × 250 mm, 5 μm) chromatographic columns were compared, and finally the peaks obtained from the Agilent ZORBAX SB-Aq C18 (2.1 × 100 mm, 3.5 μm) column were found to have better separation and higher response than those of the other columns. Selection of mobile phase: Since sphingosine is highly polar and basic, adding different proportions of formic acid or ammonium formate to the mobile phase can improve the peak shape and promote ionization. The highest response was obtained when 0.1% formic and 5 mmol/L ammonium formate were added simultaneously, and the peak shape of each component was good. Exploring the elution procedure: The five sphingolipids were initially considered for gradient elution by reversed-phase chromatography with a high proportion of organic phases due to their close structures and polarities. After multiple comparisons adjustment, it was found that when methanol–water (85:15, *v/v*) (water and methanol containing 0.1% formic acid, 5 mmol/L ammonium formate) was used for isocratic elution, component detection was completed in 3 min. Optimization of pretreatment methods: Since protein precipitation is simpler and more economical than liquid‒liquid extraction and solid-phase extraction, different proportions of acetonitrile-methanol and different proportions of methanol-isopropyl were used for protein precipitation in this study. The results showed that the extraction rate of each component was the highest when acetonitrile-methanol (9:1) was used, and the reconstitution of different ratios of methanol-isopropanol had little effect on the response value and peak shape of each component, so we used methanol for reconstitution. Selection of ion pairs: Since the detections are all sphingolipids and have similar structures, the fragmentation methods are similar, and the same or similar product ions can often be obtained. The standard concentration range during the experiment is shown in Table S-2, and the quality control samples prepared at the three concentration levels are shown in Table 1. The quantitative ion pairs and corresponding mass spectrometry conditions of each reference substance are shown in Table S-3 and Fig. 2, which indirectly confirms the rationality of the selection of ion pairs in the experimental results.

**Table 1:** Results of stability, accuracy, precision, recovery and matrix effect in quality control samples (*n* = 6)

Analyte	Sample	Added concentrations in QCs (ng/mL)	Matrix effect (%)	Stability (RSD, %)	Recovery (mean ± SD, %)	Intra- and Intraday accuracy (RE, %)	Intra- and Intraday precision (RSD, %)
Sphinganine (d16:0)	Brain	3 2000 4000	108.1 98.3 94.0	11.2 8.4	*L:83.6* ± *4.2* *M:88.5* ± *3.6* *H:82.0* ± *6.1*	97.8–103.1	3.6–5.8
	Blood	3 1000 3000	102.9 96.8 106.8	4.1 4.3	*L:*90.8 ± 4.9 *M:*82.7 ± 3.5 *H:*83.2 ± 7.6	97.3–99.9	1.4–11.9
	Cell	3 2400 3600	98.0 93.8 110.4	5.8 9.8	*L:*91.2 ± 5.8 *M:*92.8 ± 4.1 *H:*80.5 ± 7.4	98.7–100.9	3.3–7.6
S1P	Brain	10 200 400	98.9 99.6 98.2	10.9 4.7	*L:*90.2 ± 2.1 *M:*88.3 ± 4.5 *H:*87.2 ± 1.6	94.5–109.6	5.4–8.3
	Blood	10 125 375	100.4 102.2 92.3	7.2 6.7	*L:*83.4 ± 2.7 *M:*93.3 ± 4.1 *H:*91.5 ± 3.4	100.1–101.8	3.1–7.4
	Cell	10 36 54	105.1 102.0 98.6	10.9 8.3	*L:*102.4 ± 5.6 *M:*87.8 ± 2.4 *H:*94.1 ± 3.1	101.2–103.8	2.6–4.8
Sphinganine (d18:0)	Brain	3 8000 16,000	99.6 92.8 98.7	7.1 5.6	*L:*85.1 ± 1.9 *M:*90.8 ± 2.0 *H:*83.7 ± 1.8	96.1–108.3	6.1–10.2
	Blood	3 5000 15,000	106.5 95.9 95.1	12.4 10.2	*L:*91.4 ± 2.2 *M:*84.7 ± 4.6 *H:*96.6 ± 4.2	99.4–104.8	6.5–9.0
	Cell	3 6000 9000	95.3 94.3 100.9	4.8 7.5	*L:*83.1 ± 6.8 *M:*92.3 ± 1.4 *H:*87.2 ± 5.7	99.4–99.9	2.1–6.2
Phytosphingosine	Brain	3 4000 8000	105.7 105.4 93.2	7.8 8.1	*L:*89.7 ± 3.6 *M:*91.7 ± 2.1 *H:*92.3 ± 4.0	92.3–107.4	4.6–9.1
	Blood	3 5000 15,000	100.4 95.7 94.1	4.6 7.0	*L:*91.4 ± 1.2 *M:*83.4 ± 2.8 *H:*97.4 ± 6.4	98.7–103.9	2.1–11.8
	Cell	3 3000 4500	101.7 103.5 100.9	9.6 5.9	*L:*81.6 ± 2.3 *M:*91.8 ± 3.7 *H:*90.1 ± 5.4	97.9–99.3	2.8–4.9

#### Method validation

**Fig. 3:**
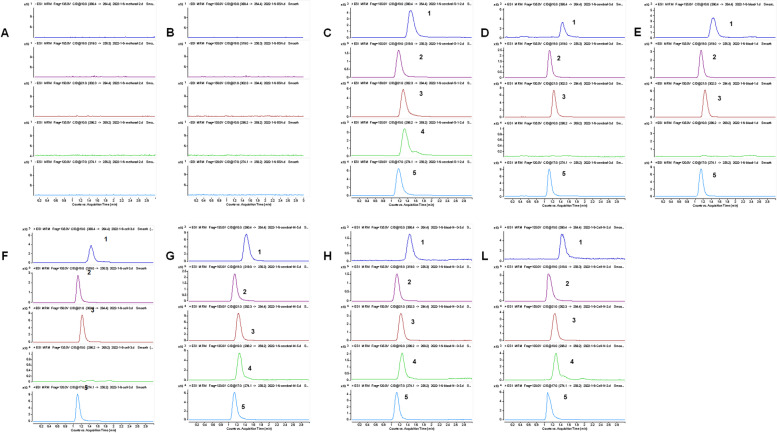
Chromatograms of sphingolipids and internal standard. **A** Methanol **B** 4% BSA **C** Standard solution (dissolved in methanol) **D** Brain tissue sample (without internal standard) **E** Serum tissue sample (without internal standard) **F** Cell sample (without internal standard) **G** Brain tissue sample (with internal standard) **H**. Serum sample (with internal standard) L. Cell sample (with internal standard). 1. Sphingosine-1-phosphate; 2. Phytosphingosine; 3. Sphinganine (d18:0); 4. Sphingosine (d17:1); 5. Sphinganine (d16:0)

A series of experiments including specificity, linear range, precision, accuracy, stability, matrix effect, and sample recovery for sphingolipid quantification validated the proposed method. Figure 3 reveals a good separation of each component with no interference impurity peak nearby. The retention times were as follows: sphinganine (d16:0) 1.14 min, sphingosine (d17:1) 1.26 min, sphinganine (d18:0) 1.24 min, phytosphingosine 1.14 min, and sphingosine-1-phosphate 1.44 min. Calibration curves for the components showed good linearity with correlation coefficients(*R*^2^) ≥ 0.993 (listed in Table S-1). The LODs and LOQs of the components were within the range of 0.5–2 ng and 1–4 ng, respectively. The results suggested the good precision and accuracy of the method (as shown in Table 1): the RSD of detection precision was 1.4 ~ 11.9%, and the RE of accuracy was 92.3 ~ 109.6%, which showed that the determination of the content was accurate and reliable. When the short-term stability, long-term stability, and freeze‒thaw stability of the samples and reference stock solution were analysed, the RSDs were 4.1 ~ 12.4% and 6.5%, respectively, which indicated that the biological samples and the stock solution had good stability under various conditions. The matrix effects obtained by the high, medium, and low concentrations of the three biological samples ranged from 92.8% to 110.4% and showed that the RSD was less than 15%, and the spiked recoveries were 80.5–102.4%, which met the methodological requirements for the analysis of biological samples.

### Comparison of four sphingosine contents in three biological samples

**Fig. 4:**
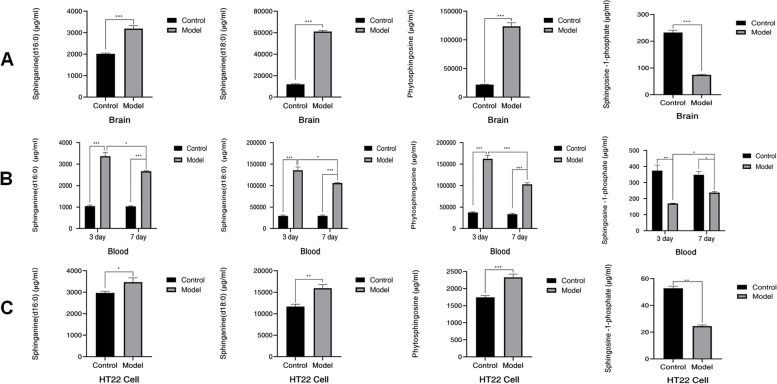
Changes in the contents of sphingosine-1-phosphate, phytosphingosine, sphinganine (d18:0), and sphinganine (d16:0) in the serum (**A**) and brain tissue (**B**) of SD rats in the normal and pMCAO groups and HT22 cells (**C**) in the normal and OGD/R groups (control, *n* = 6; model, *n* = 6). The data of each experiment are expressed as the mean ± standard deviation. Note. Compared with the normal or model group, *P < 0.05, **P < 0.01, ***P < 0.001

Comparison of the contents of four sphingosines in three biological samples (Table S-1 and Fig. 4): Given the in vitro findings, we obtained the following results: compared with the normal state, except for the content of S1P (*P* < 0.01), which was significantly decreased in HT22 cells in the OGD/R state, the phytosphingosine (*P* < 0.001), sphinganine (d18:0) (*P* < 0.01) and sphinganine(d16:0) (*P* < 0.05) contents were significantly increased. The results were similar in the in vivo experiments: compared with those in the brain tissue of SD rats in the normal group, the contents of phytosphingosine (*P* < 0.001), sphinganine (d18:0) (*P* < 0.001), and sphinganine (d16:0) (*P* < 0.001) were significantly increased, while that of S1P (*P* < 0.001) was significantly decreased in the model group. Compared with the SD rats in the normal state, the S1P content in the serum collected from the rats in the pMCAO group decreased significantly (*P* < 0.01 on the third day/*P* < 0.05 on the seventh day), while the contents of phytosphingosine (*P* < 0.001), sphinganine (d18:0) (*P* < 0.001) and sphinganine (d16:0) (*P* < 0.001) were significantly increased. The S1P content (*P* < 0.05) was increased in the serum collected on the seventh day compared to the third day in the pMCAO group rats, while phytosphingosine (*P* < 0.001), sphinganine (d18:0) (*P* < 0.05), and sphinganine (d16:0) (*P* < 0.05) were decreased. The serum collected on the seventh day of the normal group of rats showed no significant change compared with the serum collected on the third day. The results of further analysis clearly showed regular differences between the groups.

## Discussion

Lipidomics is an important branch of metabolomics, that aims to comprehensively study the lipid content in biological samples and to explore the metabolic profiles and changes in endogenous lipids under different states and interventions. The lipid composition is complex and can be divided into eight categories according to the skeleton structure, such as fatty acids, glycerolipids, glycerophospholipids, and sphingolipids [24]. Lipid profiles were significantly correlated with visceral obesity [25]. Similarly, disorders of lipid metabolism are closely related to neurodegenerative diseases such as cerebral ischemia and Alzheimer's disease [26, 27]. Lipids may be useful in assessing the prognosis and staging of disease severity in neurodegenerative diseases such as cerebral ischemia.

Sphingolipids are the main lipids in the central nervous system. They are abundant in the brain and highly expressed in myelin. The hydrolysis of sphingolipids can produce ceramide (Cer), ceramide-1-phosphate, sphingosine, and phosphorylated sphingosine S1P [28]. Dietary sphingolipids can also be hydrolyzed into ceramides and sphingosines for absorption [29]. Sphingolipids play an important role in the stabilization and transmission of synapses, regulation of signaling pathways, and neuronal survival. Sphingolipidomics is sphingolipid-targeted lipidomics, which mainly explores the changes in the structure and metabolism of sphingolipids [30]. Sphingolipidomics research has made important findings in the pathogenesis of ischemic stroke: plasma levels of brain-specific SLs may serve as effective biomarkers of cerebral microvascular disease [31]. Stroke animal models show that sphingolipid content changes with acute cerebral ischemic injury [32]. Ceramide and S1P play an important role in neuronal signal transduction [33]. However, there are few studies on cells and animal models from the perspective of sphingolipidomics to reveal the relationship between absolute changes in endogenous sphingolipids and stroke. These representative endogenous sphingolipid components may be potential therapeutic targets for ischemic stroke.

### The significant decrease in the S1P level after modeling is closely related to cerebral ischemia

S1P is the most characteristic bioactive sphingolipid in cells, mainly from erythrocytes, platelets, and endothelial cells [34]. S1P regulates cell homeostasis, activates glial cells, and plays an important role in inflammation, oxidation and angiogenesis [35–37]. Moreover, S1P has recently emerged as an integral player in coordinating lymphatic transport and maintaining endothelial cell barrier integrity by activating multiple cognate receptors, such as S1PR1, to resist stroke injury [38]. Clinical studies have shown that the regulation of S1P homeostasis plays a key role in cerebrovascular disease and vascular dementia, demonstrating that S1P is associated with cognitive deficits in cerebrovascular disease [39]. By improving mitochondrial dysfunction and other pathways, S1P can regulate apoptosis and the inflammatory response in the cerebral ischemic area to alleviate brain injury [40]. In addition, the plasma S1P level in stroke patients is closely related to the collateral circulation status, and the lower the S1P level is, the worse the stroke prognosis, indicating that S1P may be a potential stroke collateral circulation status and prognosis biomarker [41, 42].

Research showed that S1P levels in serum, brain tissues and HT22 cells in the model group were significantly lower than those in the control group, which is consistent with the trend of lowering S1P levels in the serum of clinical patients with acute cerebral ischemia [43]. However, the level of S1P in serum on the seventh day was higher than that on the third day. Combined with the changes in the body weight of the rats plateauing over time, it is speculated that a large number of cells were damaged in the acute phase of cerebral ischemia, resulting in the reduction of S1P production and its receptors, consumption increases, so the S1P content drops sharply. The ischemic area in the stable and convalescent phases restores the synthesis and release of S1P by participating in the process of cell-autonomous SIP supply, gradually restoring blood‒brain barrier function, microvascular patency, and blood supply to hypoperfused brain tissue through collateral anastomosis, which can lead to increased S1P levels. Based on the trend of S1P changes, it is hypothesized that S1P could be a potential biomarker for ischemic stroke based on its neuroprotective effect of improving inflammation and promoting angiogenesis.

### Except for S1P, sphingosine levels were significantly elevated after modeling and correlated with cerebral ischemia severity

Sphingosine produces S1P under the action of sphingosine kinases (SphKs), and S1P phosphatase can convert S1P back to sphingosine [44]. Sphingosine acts as an intracellular signaling lipid, increases neurotransmitter release in neurons, and affects the heterologous and homologous fusion of organelles, which may underlie sphingosine-derived lipids in the treatment of neurodegenerative diseases. In humans, phytosphingosine linked to an α-hydroxylated acyl chain is more abundant than the same linked sphingosine [45]. Phytosphingosine can be used as a potential predictor to distinguish cerebral thrombosis caused by different origins [46]. Sphingolipids such as dihydro-sphingosine change with hypoxia in brain endothelial cells [47]. Sphingolipids such as dihydrosphingosine, sphingosine, and S1P have been associated with neurodegenerative-related neurological diseases and may be involved in the pathophysiological mechanism of stroke [48].

The results showed that the levels of sphinganine (d16:0), sphinganine (d18:0), and phytosphingosine were increased in the model group compared with the control group, and the changes were related to the metabolic balance consisting of ceramide, sphingosine, and S1P. In recent years, studies have shown that ceramide, sphingosine kinase, and S1P can be involved in multiple links in the process of cerebral ischemia, suggesting that they can be used as potential targets for the treatment of cerebral ischemia [49]. The changes in sphinganine (d16:0), sphinganine (d18:0), phytosphingosine, and S1P content were integrated and analyzed to reveal their trends over time and dynamic balance to explore the severity and prognosis of stroke.

### Combination of the background deduction method and matrix substitution method for LC‒MS/MS analysis

In this study, an LC‒MS method for the analysis of the content of four sphingolipids in ischemic stroke rats and OGD/R HT22 cells was established by combining the background subtraction method and the matrix substitution method.

The targeted qualitative and quantitative analysis of endogenous components such as sphingolipids has become a new approach for disease research. Due to the complexity of sphingolipid composition, various advanced analytical instruments and analytical methods are applied in the detection of sphingolipids. Early studies, such as Bazán [50], showed that changes in sphingolipid content could be detected after brain injury. With the advent of LC‒MS, the rapid and precise identification and quantification of endogenous lipids can be achieved. Brusatori M [51]analyzed the content of ceramide in human visceral and subcutaneous adipose tissue by LC‒MS and found that ceramide was correlated with diabetes and gender. Cho HE [18] achieved the distinction and quantification of C18-L-threo-sphinganine and the diastereomer C18-D-erythro-sphinganine in human plasma by HILIC‒LC‒MS. Brogden [52] conducted qualitative and quantitative studies on sphingosine and sphinganine by chemical derivatization and liquid phase-fluorescence detection. Sphingolipids are structurally modified by chemical derivatization technology, which improves chromatographic resolution, detection sensitivity, and selectivity. In recent years, with the improvement of the analytical methods of sphingolipids, the study of disease has been more in depth. The high resolution, mass accuracy and mass axis stability of the LTQ-Orbitrap XL were used to scan brain tissue slices. The lipid spectra obtained for each pixel are transformed to color-coded intensity maps of hundreds of lipid species [53]. This analytical method can detect the multiplicity of lipid complex species present in the brain and the distribution of lipids in the brain. However, this method is mostly applicable to the transformation process from disease research to clinical application, which is relatively limited to serum samples and in vitro experiments. Research describes two liquid chromatography tandem mass spectrometry methods for component separation through different chromatographic columns, isotope-labeled internal standards, and multipoint calibration curves to quantify four lysosphingolipid species [54]. This method can detect the change trend of plasma lysosphingolipids, but cannot realize the absolute quantification of this component.

Although most LC‒MS studies have identified associations between specific lipids and disease progression, they have not yet been translated into clinically useful diagnostic tests. Part of this gap may be explained by insufficient depth of the lipidomic profiles in many studies and a lack of absolute quantification [55]. Based on the identification and semiquantitative detection of endogenous lipid metabolites in clinical complex biological matrix samples, it is necessary to analyze their absolute quantification. Liquid‒liquid extraction and solid-phase extraction are commonly used extraction and purification methods for lipids. Chloroform–methanol-water, chloroform–methanol, butanol-methanol, and methyl tert-butyl ether-methanol–water are commonly used as extraction reagents [56]. In this study, the lipids of rat brain and HT22 cells were extracted by chloroform–methanol, and the absolute contents of sphinganine (d16:0), sphinganine (d18:0), phytosphingosine, and S1P were measured by background deduction. The absolute quantitative analysis of rat blood lipids was carried out by using bovine serum albumin (BSA) to simulate a blank serum matrix, which was named the matrix substitution method. Derivatization can be used to replace endogenous components in cells for detection [57]; however, it may not be suitable for absolute quantification due to incomplete response or attrition. Brain tissue is more complex than serum. It is feasible to use 4% BSA as a substitute for brain tissue, but it is not optimal for the absolute quantification of endogenous components such as sphingolipids.

### Study strengths and limitations

Reported studies suggested that monkey brain homogenate was used instead of human brain homogenate for matrix substitution and combined with stable isotope labeling of endogenous components to complete analyte substitution for the quantification of endogenous 2-hydroxyglutarate in human brain tumors by LC‒MS [16]. Monkey brain homogenates are scarce, not easy to obtain, and cumbersome and costly to operate. Stable isotopes are almost identical in structure to the endogenous biomolecules to be analyzed and have identical physicochemical properties, making them the most ideal, accurate, and reliable method for absolute quantitative analysis. However, due to the difficulty and high cost of synthesizing stable isotope-labeled internal standard compounds, they cannot be widely used in basic research and clinical practice. Studies have found that electrospray ionization mass spectrometry (ESI–MS) can be more sensitive and specific for the detection of lipid stable isotope-labeled substrates without the process of lipid hydrolysis and derivatization [58]. In this experiment, the background subtraction method was used to detect the endogenous components of rat brain tissue and cells, BSA was used to simulate blank serum for matrix substitution, and accurate and rapid quantification was achieved by LC‒MS. However, background subtraction requires the selection of an appropriate number of typical samples. Consideration of sample size and possible selection bias improves the representativeness of the sample and the reliability of the method. Overall, this method does not need stable isotopes and structural derivatization, which can be more convenient and widely applied to the analysis of lipid metabolism in clinical samples, and provides a reference for the rapid and accurate determination of endogenous biomarker content related to clinical diseases.

## Conclusions

In summary, the LC‒MS/MS quantitative detection method established with sphingosine (d17:1) as the internal standard, BSA as a simulated blank serum matrix for matrix replacement, and multiple batches of normal brain tissue and cell samples for background subtraction provides a reference for the detection of a series of endogenous components, such as sphingolipids. The levels of endogenous sphingolipids such as S1P in animal and cell models show that the content of each component is closely related to the occurrence and development of stroke, which provides a basis for the link between the severe staging of stroke and the relative trend of sphingolipids such as S1P. S1P, phytosphingosine, sphingosine (d18:0), and sphingosine (d16:0) may be potential predictors of stroke injury severity and prognosis. Through targeted analysis of endogenous sphingolipid trends, it is possible to reveal the role of sphingolipids in stroke and provide evidence for the discovery of potential therapeutic targets for stroke.

## Supplementary Information

**Additional file 1: Supplementary Table 1.** Linear relationship and sample concentration range of serum, brain tissue, and cell components (normal A is the normal serum taken on the third day, normal B is the normal serum taken on the seventh day; the same for the model group) (This concentration ranges from 100μL brain tissue homogenate, 100μL serum, 1×10^6^ cells).

**Additional file 2: Supplementary Table 2.** Concentration ranges of sphinganine (d16:0), sphingosine (d17:1), sphinganine (d18:0), phytosphingosine, and sphingosine-1-phosphatein in 100μL brain tissue homogenate, 100μL serum, and 1×10^6^ cells.

**Additional file 3: Supplementary Table 3.** Mass spectrum parameters of MRM of five kinds of sphingolipids.

## Data Availability

The data (raw and processed) used in the current study will be available from the corresponding author on reasonable request.

## Abbreviations

OGD/R: Oxygen-glucose deprivation
pMCAO: Permanent middle cerebral artery occlusion
S1P: Sphingosine-1-phosphate
BHT: 2,6-Di-tert-butyl-4-methylphenol
MRM: Multiple reaction monitoring
ESI: Electrospray ionization
CID: Collision-induced dissociation
QC: Quality control
LQC: Low quality control
MQC: Medium quality control
HQC: High quality control
OD: Optical density
LOD: Low optical density
LLOQ: Lower limit of quantification
IS: Internal standard
SD: Standard deviation
RSD: Relative standard deviation
RE: Relative error
SphKs: Sphingosine kinases
ESI–MS: Electrospray ionization mass spectrometry
