# Supplementary material for: Determination of endogenous sphingolipid content in stroke rats and HT22 cells subjected to oxygen-glucose deprivation by LC‒MS/MS
Source: Lipids Health Dis. 2023 Jan 25;22:13. doi: 10.1186/s12944-022-01762-3 (PMC9878918; doi:10.1186/s12944-022-01762-3)
Supplement: Supplementary file 1 — Additional file 1: Supplementary Table 1. Linear relationship and sample concentration range of serum, brain tissue, and cell components (normal A is the normal serum taken on the third day, normal B is the normal serum taken on the seventh day; the same for the model group) (This concentration ranges from 100μL brain tissue homogenate, 100μL serum, 1×10^6^ cells). [file 12944_2022_1762_MOESM1_ESM.docx]

| Sample |  | Sphingosine -1-phosphate | | | phytosphingosine | | | Sphinganine(d18:0) | | | Sphinganine(d16:0) | | |
| --- | --- | --- | --- | --- | --- | --- | --- | --- | --- | --- | --- | --- | --- |
|  | Group | Formula A | Formula C | C（ng/ml) | Formula A | Formula C | C（ng/ml) | Formula A | Formula C | C（ng/ml) | Formula A | Formula C | C（ng/ml) |
| Brain | nomal | Y=0.0198X+0.7817 | Y=0.0198X-0.00004 | 232.82±20.97 | Y=0.0078X+38.336 | Y=0.0078X+0.0004 | 21767.72±1832.32 | Y=0.0015X+5.404 | Y=0.0015X-0.00002 | 11965.26±1303.82 | Y=0.0066X+9.9582 | Y=0.0066X+0.0653 | 2019.03±79.49 |
|  | model | R^2^=0.9968 | R^2^=0.9968 | 74.49±5.08 | R^2^=0.9994 | R^2^=0.9994 | 123622.43±15557.52 | R^2^=0.9989 | R^2^=0.9989 | 61089.98±2682.43 | R^2^=0.9991 | R^2^=0.999 | 3186.97±348.64 |
| Blood | nomal A | Y=0.0004X+0.0027 | | 374.80±34.51 | Y=0.0004X+0.0275 | | 37693.67±3765.05 | Y=0.0017X+0.0874 | | 29744.33±4320.48 | Y=0.002X+0.0697 | | 1046.46±131.11 |
|  | model A |  |  | 170.26±1.72 |  |  | 162599.42±17736.51 |  |  | 135940.37±18185.80 |  |  | 3372.43±426.21 |
|  | nomal B | R^2^=0.9998 | | 349.00±20.25 | R2=0.9965 | | 33788.38±4767.62 | R^2^=0.9982 | | 29811.66±4365.08 | R^2^=0.9936 | | 1036.27±46.79 |
|  | model B |  |  | 237.65±7.13 |  |  | 102990.94±8846.97 |  |  | 105919.21±3409.34 |  |  | 2668.35±100.34 |
| Cell | nomal | Y=0.0052X+0.0229 | Y=0.0052X-4E-06 | 52.20±3.75 | Y=0.0022X+57.036 | Y=0.0022X-0.0004 | 1747.80±125.54 | Y=0.0016X+11.453 | Y=0.0016X-0.00006 | 11654.23±1395.23 | Y=0.0026X+14.06 | Y=0.0026X+0.0004 | 2967.78±201.37 |
|  | model | R^2^=0.9951 | R^2^=0.9951 | 24.97±2.14 | R^2^=0.9992 | R^2^=0.9992 | 2330.36±226.89 | R^2^=0.9937 | R^2^=0.9937 | 15934.62±2120.73 | R^2^=0.9943 | R^2^=0.9943 | 3470.53±489.36 |

**Supplementary Table 1.** Linear relationship and sample concentration range of serum, brain tissue, and cell components (normal A is the normal serum taken on the third day, normal B is the normal serum taken on the seventh day; the same for the model group) (This concentration ranges from 100μl brain tissue homogenate, 100μl serum, 1×10^6^ cells)
