# Supplementary material for: Determination of endogenous sphingolipid content in stroke rats and HT22 cells subjected to oxygen-glucose deprivation by LC‒MS/MS
Source: Lipids Health Dis. 2023 Jan 25;22:13. doi: 10.1186/s12944-022-01762-3 (PMC9878918; doi:10.1186/s12944-022-01762-3)
Supplement: Supplementary file 2 — Additional file 2: Supplementary Table 2. Concentration ranges of sphinganine (d16:0), sphingosine (d17:1), sphinganine (d18:0), phytosphingosine, and sphingosine-1-phosphatein in 100μL brain tissue homogenate, 100μL serum, and 1×10^6^ cells. [file 12944_2022_1762_MOESM2_ESM.docx]

**Supplementary Table** **2**. Concentration ranges of sphinganine (d16:0), sphingosine (d17:1), sphinganine (d18:0), phytosphingosine, and sphingosine-1-phosphatein in 100μl brain tissue homogenate, 100μl serum, and 1×10^6^ cells.

| Sample | sphinganine(d16:0) | sphingosine (d17:1) | sphinganine(d18:0) | phytosphingosine | S1P |
| --- | --- | --- | --- | --- | --- |
| Blood | 20ng~4μg | 100ng | 100ng~20μg | 100ng~20μg | 2.5ng~500ng |
| Brain | 50ng~5μg | 100ng | 100ng~20μg | 100ng~10μg | 5ng~500ng |
| Cell | 200ng~4μg | 100ng | 500ng~10μg | 25ng~5μg | 3ng~60ng |
