# Supplementary material for: Determination of endogenous sphingolipid content in stroke rats and HT22 cells subjected to oxygen-glucose deprivation by LC‒MS/MS
Source: Lipids Health Dis. 2023 Jan 25;22:13. doi: 10.1186/s12944-022-01762-3 (PMC9878918; doi:10.1186/s12944-022-01762-3)
Supplement: Supplementary file 3 — Additional file 3: Supplementary Table 3. Mass spectrum parameters of MRM of five kinds of sphingolipids. [file 12944_2022_1762_MOESM3_ESM.docx]

### Supplementary Table 3. Mass spectrum parameters of MRM of five kinds of sphingolipids

| Analyte | Precursor Ion | Product Ion | Fragmentor | CID | Polarity |
| --- | --- | --- | --- | --- | --- |
| Sphinganine(d16:0) | 274.1 | 256.2 | 120 | 17 | Positive |
| Sphinganine(d18:0) | 302.3 | 284.4 | 150 | 21 | Positive |
| Phytosphingosine | 318.0 | 256.3 | 105 | 19 | Positive |
| S1P | 380.4 | 264.4 | 135 | 10 | Positive |
| Sphingosine(d17:1) | 286.2 | 268.2 | 135 | 10 | Positive |
